# Effect of Sourdough–Yeast Co-Fermentation on Physicochemical Properties of Corn Fagao Batter

**DOI:** 10.3390/foods13172730

**Published:** 2024-08-28

**Authors:** Qianhui Yang, Yingguo Lyu, Zhenhua Wu, Xueqin Li, Kunlun Liu

**Affiliations:** 1 College of Food Science and Engineering, Henan University of Technology, Zhengzhou 450001, China; qianhui0206@163.com (Q.Y.); wuzhenhua1028@163.com (Z.W.); xueqin1216@sina.cn (X.L.); knlnliu@126.com (K.L.); 2 Henan Province Wheat-Flour Staple Food Engineering Technology Research Centre, Zhengzhou 450001, China

**Keywords:** sourdough, corn Fagao, batter, co-fermentation, starch

## Abstract

Fagao is one of China’s traditional gluten-free staple foods made with rice or corn flour. Corn Fagao prepared by co-fermentation with sourdough and yeast exhibits better quality and less staling compared to traditional yeast-fermented Fagao. The physicochemical properties of corn Fagao batter during sourdough–yeast co-fermentation were investigated. The results showed that compared with yeast fermentation, the gas production and viscosity of the batter increased with co-fermentation. The co-fermented batter showed a higher hydrolysis of starch and less amylose content. The integrity of starch granules in the co-fermented batter was damaged more seriously, and the crystallinity and short-range ordered structure were less than in the yeast-fermented batter, even though the crystal structure type of starch did not obviously change. The peak viscosity, minimum viscosity, final viscosity, decay value, and recovery value of the corn batter were reduced by co-fermentation, which improved the thermal stability of the batter and slowed down the aging. Co-fermentation also resulted in a more pronounced reduction in protein subunit content than yeast fermentation. The changes in the physicochemical properties of the corn Fagao batter help explain the improvement in quality of corn Fagao made from the co-fermentation method and may provide theoretical references for co-fermentation with sourdough and yeast to other gluten-free foods.

## 1. Introduction

The production of sourdough is typically achieved through the fermentation of microorganisms, water, cereals, pseudo cereals, or legumes. It is primarily employed in the manufacture of fermented flour products [1]. The microbial composition of different sourdoughs varies, with the most dominant strains being Lactic acid bacteria and yeasts, with Lactic acid bacteria dominating and playing a positive role in the quality of sourdough-fermented products [2]. The incorporation of sourdough into fermented dough products can enhance their rheological and functional properties. This is achieved through the action of extracellular polysaccharides, enzymes, organic acids, and carbon dioxide produced during the growth and metabolism of the fermenting microorganisms [3]. Sourdough fermentation is known to not only have a positive effect on the quality and shelf-life of wheat bread but also improve its flavor and aroma [4,5]. For instance, the utilization of sourdough in the production of gluten-free bread serves to reduce product hardness and increase bread-specific volume [6]. Mantzourani et al. indicated that sourdough based on *Lactobacillus paracasei* not only extends the shelf-life of the bread but also enhances its flavor and aroma [7]. In addition, the presence of sourdough not only alters the texture of bread but also enhances its nutritional value and provides beneficial effects on health [8,9,10]. Some positive effects have been almost definitively proven. The action of organic acids produced during sourdough fermentation results in a reduction in the degree of starch pasting, which in turn leads to a notable decline in the starch digestibility hydrolysis index and the predicted glycemic index of the bread. This has the beneficial effect of improving blood sugar levels in humans [11]. In another study, the content of the anti-nutritional factor phytic acid in whole-wheat bread reduces the phytase produced by sourdough fermentation, which promotes the digestion and absorption of nutrients [12].

**Figure 1:**
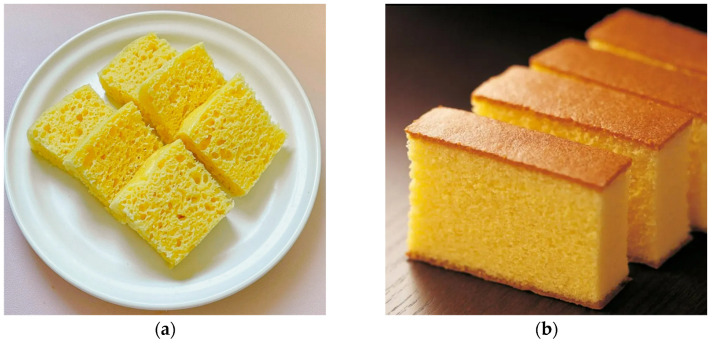
Fagao and sponge cake. (**a**) Fagao; (**b**) sponge cake.

Fagao (steamed sponge cake) is one of China’s traditional staple foods, prepared with grains such as rice and corn through a process of batter mixing, molding, fermentation, steaming, and decorating. The Chinese pronunciation of Fagao has the meaning of wealth and promotion. It has become an essential pastry for Chinese New Year festivals [13]. Fagao is similar to sponge cake, but there are differences between the two. Figure 1 presents a comparison of the two products. While both Fagao and sponge cake are batter products with a uniform dispersion of holes, soft textures, and other characteristics, there are notable differences in their raw materials, leavening ingredients, and cooking methods. Unlike sponge cake, which is cooked by baking, Fagao is steamed to achieve its desired texture. Sponge cake is usually made with cake flour, rich in a formula containing eggs and oil, which helps form a porous structure. Fagao is usually made with gluten-free ingredients without eggs and oil. Additionally, Fagao requires fermentation, a process that sponge cakes do not undergo. The process of fermentation is of great significance in the production of Fagao. It is responsible for imparting a soft and elastic texture, a uniform honeycomb pore structure, and a distinctive post-fermentation aroma to Fagao, which is highly regarded by consumers. Yeast is the common starter used in Fagao fermentation.

As the largest planted crop in the world, corn is a common ingredient in Fagao. Corn is rich in nutrients like starch, protein, fat, and linoleic acid [14]. Corn Fagao is an excellent gluten-free staple for individuals with celiac disease. However, corn Fagao is susceptible to staling, which ultimately results in a reduction in its overall quality. Our previous study showed that corn Fagao prepared by co-fermentation with sourdough and yeast exhibits better appearance, internal structure, texture, and flavor compared to traditional yeast-fermented Fagao [15]. Thus, the drawback of Fagao may be improved by co-fermentation with a variety of fermented microorganisms. Among the available studies, there are fewer studies on the properties of corn Fagao batter during co-fermentation.

The present study aimed to investigate the corn Fagao batter characteristics and physicochemical properties of corn starch molecules during co-fermentation with sourdough and yeast. The findings aim to provide theoretical references for co-fermentation with sourdough and yeast to improve the quality of corn-based fermented foods.

## 2. Materials and Methods

### 2.1. Materials

The lyophilized powder of *Lactobacillus plantarum* (10^11^ CFU/g) was procured from Shandong Zhongke Jiayi Biotechnology Co., Ltd. (Wefang, China). The yeast was purchased from Angel Yeast Co., Ltd. (Yichang, China). Corn flour and sugar were obtained from local commercial markets. All the procured chemicals were analytical grade.

### 2.2. Preparation of Sourdough

A basic sourdough recipe consisting of corn flour (100 g), *Lactobacillus plantarum* powder (1 g), and water (120 g) was used to prepare the sourdough. The ingredients were combined in a mixing bowl. Following this, the dough was proofed in a fermentation cabinet (LHS-100CL, Yiheng Scientific Instrument Co., Shanghai, China) at 30 °C and 75% relative humidity for 10 h. This proofed dough was designated as sourdough.

### 2.3. Preparation of Corn Fagao Batter

The corn Fagao batter was prepared according to the methodology reported by Wu et al. [15] with slight modifications. The sourdough–yeast co-fermented corn Fagao batter recipe, consisting of corn flour (200 g), sugar (24 g), water (120% of corn flour), and starter (10% sourdough and 1% yeast), was used to prepare the co-fermentation corn Fagao batter. The ingredients were combined in a mixing bowl for 3 min. Following this, the corn Fagao batter was proofed in a fermentation cabinet at 30 °C and 75% relative humidity for a while. The fermentation times of 0, 15, 30, and 45 min of the co-fermented sourdough and yeast were abbreviated as SY-0, SY-15, SY-30, and SY-45, respectively. The control group consisted of starter (1% yeast) fermentation only, which was recorded as YY-0, YY-15, YY-30, and YY-45. Batter samples were obtained at each of the specified time points for subsequent analysis. Part of the corn Fagao batter with different fermentation conditions was freeze-dried by a vacuum freeze-drier (77530-6L, Labconco Equipment Co., Kansas City, MO, USA), milled into powder which was then sifted through an 80 mesh sieve, and stored in a sealed container for further analysis.

### 2.4. Determination of pH and Total Titratable Acidity (TTA)

The pH and TTA of the corn Fagao batter with different fermentation conditions were measured by the modified method by Nagihan et al. [16]. Up to 10 g of corn Fagao batter with different fermentation conditions was homogenized with 100 mL of distilled water, and the pH was measured using a pH meter (PHS-25, Qiwei Instrument Co., Hangzhou, China). The aforementioned mixture was then titrated with a 0.1 mol/L NaOH solution until the pH reached 8.6. The volume of NaOH solution required to achieve this endpoint was recorded as the total acidity (TTA).

### 2.5. Determination of Reducing Sugar Content

The method of testing the reducing sugar content as described by Li et al. was used with a brief modification [17]. Up to 10 g of corn Fagao batter with different fermentation conditions was homogenized with 100 mL of distilled water. The sample solution consisted of 100 mL of distilled water homogenized with 10 g of corn Fagao batter with different fermentation conditions. The reducing sugar content of the samples was quantified using the 3,5-Dinitrosalicylic acid spectrophotometric method (7230G, Ningbo Shunyu Instrument Co., Ningbo, China).

### 2.6. Determination of Specific Gravity

The method of testing the specific gravity as described by Charlotte et al. was used with a brief modification [18]. Following fermentation, the density of each sample was determined in triplicate, as the ratio of the batter weight to the water weight filled in a standard container.

### 2.7. Determination of Viscosity

The viscosity of the corn Fagao batter with different fermentation conditions was determined using a viscometer (SNB-1, Tianmei Balance Instrument Co., Shanghai, China), where the spindle revolved at 6 rpm in 5 g of corn Fagao batter with the different fermentation conditions (adapted from a method of Cui et al., with slight modifications [19]).

### 2.8. Determination of Amylose Content

The amylose content of the dried corn Fagao batter sample was calculated by absorbance according to GB/T 15683-2008 [20].

### 2.9. The Microstructure of Starch Particles

A total of 0.1 g of corn Fagao batter with different fermentation conditions was dispersed in 10 mL of distilled water. The mixture was then observed and photographed using an optical microscope (E5, Ningbo Shunyu Instrument Co., Ningbo, China) at 1000 times magnification.

### 2.10. X-ray Diffraction (XRD)

A total of 20 mg of dried corn Fagao batter sample was subjected to X-ray diffraction (X’Pert PRO-XRD, PANalytical, Shanghai, China) scanning under the following conditions: The tube flow rate was 40 mA; tube pressure was 40 kV; scanning speed was 2°/min; with starting angle 5° and ending angle of 40°, respectively. Origin 2018 software (Origin Lab, Inc., Northampton, MA, USA) was used to analyze the crystalline peak area (Ac) and the total area A to determine the relative crystallinity = (Ac/A) × 100 [21].

### 2.11. Rapid Viscosity Analysis (RVA)

Up to 3 g of dried corn Fagao batter sample was placed in 25 mL of distilled water, and the lyophilized powder was stirred rapidly with a stirrer to disperse it completely. Pasting properties of the sample were evaluated according to AACC 76-21 by a Rapid Visco Analyzer (RVA-4, Newport Scientific Instruments, Inc., Shanghai, China).

### 2.12. Fourier Transform Infrared Spectroscopy (FTIR)

The method of Jiang et al. [22] was referred to, with minor modifications. The samples prepared by mixing 2 mg of dried corn Fagao batter sample and 0.2 g of KBr were scanned by an infrared absorption spectrometer (WQF-510, Riley Analytical Instruments, Inc., Beijing, China). Omnic 9.2 software was used to analysis the FTIR spectra and calculate the ratio of peak intensity at 1047/1022 cm^−1^ and 995/1022 cm^−1^.

### 2.13. SDS-Polyacrylamide Gel Electrophoresis

The method of SDS-PAGE as described by Manoukian et al. was used with a brief modification [23]. The dried corn Fagao batter samples (25 mg) were solubilized in 1 mL sample buffer, boiled for 5 min, and centrifuged at 10,000 rpm for 5 min before electrophoresis (24EN, Six One Biotechnology Ltd., Beijing, China). For samples, SDS-PAGE experiments were performed using a 4% stacking gel and 15% separating gel. The resulting supernatant (10 µL) was loaded onto the gels, and the electrophoresis was then run at 20 mA until the tracking dye reached the bottom of the gel. Then, the electrophoresis gel was dyed with Coomassie brilliant blue solution for 2 h, and the images were analyzed using Quantity One (Bio-Rad, Hercules, CA, USA) after decolorizing.

### 2.14. Statistical Analysis

All experiments were repeated three times, and the variance was analyzed using SPSS19.0 software; results expressed as mean ± standard deviation (SPSS Inc., Rockville, MD, USA). Different lowercase letters indicated significant differences between the samples (*p* < 0.05). Origin 2018 software was used to draw the figures.

## 3. Results

### 3.1. Effect of Co-Fermentation on the Properties of Corn Fagao Batter

#### 3.1.1. pH and TTA of the Corn Fagao Batter

**Figure 2:**
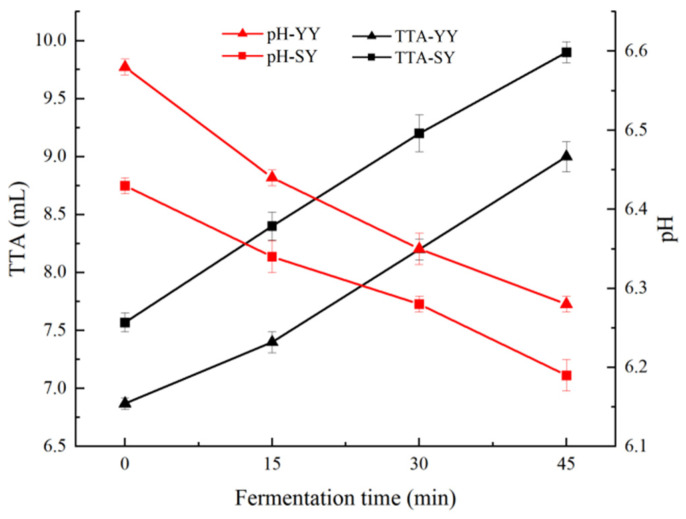
The changes in pH and TTA of corn Fagao batter.

The effects of fermentation on pH and TTA of corn Fagao batter are shown in Figure 2. The pH value represents the concentration of hydrogen ions in the corn Fagao batter, thereby reflecting the strength of acidity and alkalinity of the samples. As shown in Figure 2, the pH declined during the fermentation period in both corn Fagao batter groups. The pH of the sourdough co-fermentation group exhibited a consistently lower value than that of the yeast fermentation group. The pH of the sourdough and yeast co-fermentation was lower than that of the yeast fermentation during early fermentation might be due to lactic acid produced by the Lactic acid bacteria, which are the dominant strain in sourdough during the fermentation process [2]. As co-fermentation progresses, the acidification of the Lactic acid bacteria promotes yeast fermentation and, similarly, yeast fermentation and metabolites affect the growth and metabolism of the Lactic acid bacteria [24]. The process of co-fermentation results in the production of lactic acid, carbon dioxide, and acetic acid thus resulting in a lower pH of batter than yeast fermentation.

TTA can reflect microbial fermentation acid production; the lower the pH, the higher the TTA. As shown in Figure 2, it can be seen that the TTA of the two groups of corn Fagao batter showed an increasing trend with fermentation, and the co-fermentation group was always higher than the yeast fermentation group. This may be due to fact that the microorganisms in co-fermentation are more diverse and have a stronger acid production capacity than the yeast fermentation. The above results indicate that co-fermentation decreases the pH and accelerates the acidification of corn Fagao batter. The higher acidity of the batter facilitates the activation of amylase activity and endogenous proteases in the corn flour, which results in the formation of more soluble sugars and free amino acids, which is conducive to the improvement in the product’s flavor and texture [25].

#### 3.1.2. Reducing Sugar Content of Corn Fagao Batter

**Figure 3:**
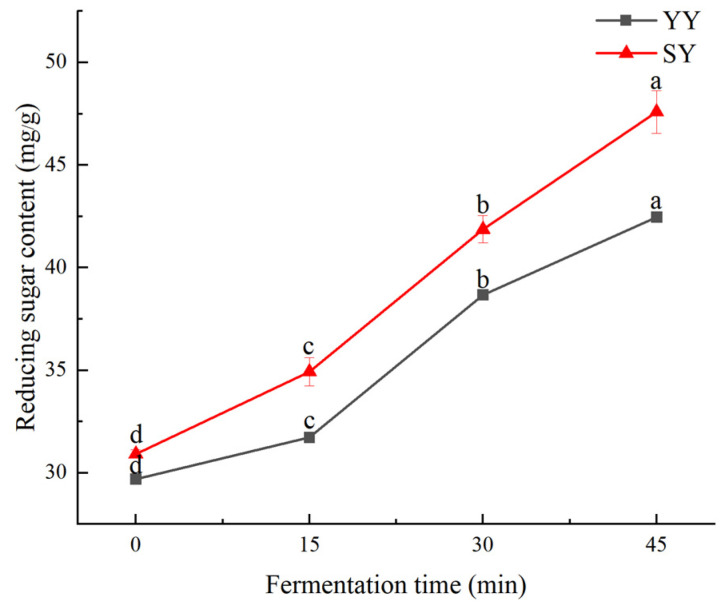
The changes in reducing sugar content of corn Fagao batter. Different lowercase letters indicate significant differences between different fermentation time at the same fermentation method (*p* < 0.05).

The generation of products by microorganisms through respiration and fermentation is inextricably linked to the metabolism of sugars in corn Fagao batter. Various enzymes and microorganisms produced during the fermentation process of corn Fagao batter convert some of the polysaccharides and oligosaccharides into reducing sugars. Consequently, the extent of fermentation in the corn Fagao batter can be quantified by measuring the reducing sugar content [26]. As shown in Figure 3, the reducing sugar content exhibits a gradual increase under the different fermentation methods with the extension of fermentation time.

At the commencement of fermentation, the reducing sugar content of the co-fermentation group was marginally higher than that of the yeast group. This suggests that the increase in reducing sugar content of the corn Fagao batter was due to the addition of sourdough. At 15 min of fermentation, the growth rate of reducing sugars was greater in the co-fermentation group than in the yeast fermentation group, indicating an increase in starch hydrolysis with the addition of sourdough. The growth rate of reducing sugar in the co-fermentation group was found to be less than that observed in the yeast fermentation group at 30 min of fermentation. This was attributed to the rapid growth of Lactic acid bacteria and yeast, which resulted in a higher rate of reducing sugar consumption, leading to a slower growth rate of reducing sugars in the co-fermentation group. At 45 min of fermentation, the reducing sugar content increased under both fermentation methods, but their growth rates were smaller than at 30 min of fermentation. At this time, the nutrients present in the corn Fagao batter had been depleted, resulting in a reduction in the overall metabolic hydrolysis and a decline in the growth rate of the reducing sugars.

Reducing sugar content in the corn Fagao batter systems depends on the dynamic equilibrium between the hydrolysis of starch by acids and enzymes to produce reducing sugars as well as the consumption of reducing sugars by microbial growth and reproduction. The reducing sugar contents showed an increasing trend during fermentation, indicating that reducing sugar produced by starch hydrolysis and microbial metabolism was higher than those consumed by microbial growth. The increased acidity helps to activate α-amylase present in grains, which converts starch to reducing sugars and increases the reducing sugar content. The higher reducing sugar content in the co-fermentation group than in the yeast fermentation group may be related to the metabolic pathways of the two different microorganisms [27]. The increase in reducing sugar content may promote the non-enzymatic browning reaction by providing a substrate for caramelization or the Maillard reaction, thereby imparting the product with an enhanced flavor and color.

#### 3.1.3. Specific Gravity of Corn Fagao Batter

Specific gravity is an important physical property of the batter, which represents the retention rate of air bubbles in the batter. The specific gravity of the batter is inversely proportional to the volume and fluffiness of the product; the lower the specific gravity of an equal mass of batter, the greater the volume of the product and the fluffier the tissue [18]. At the same time, the specific gravity can reflect the gas production of the strain utilizing sugars.

**Figure 4:**
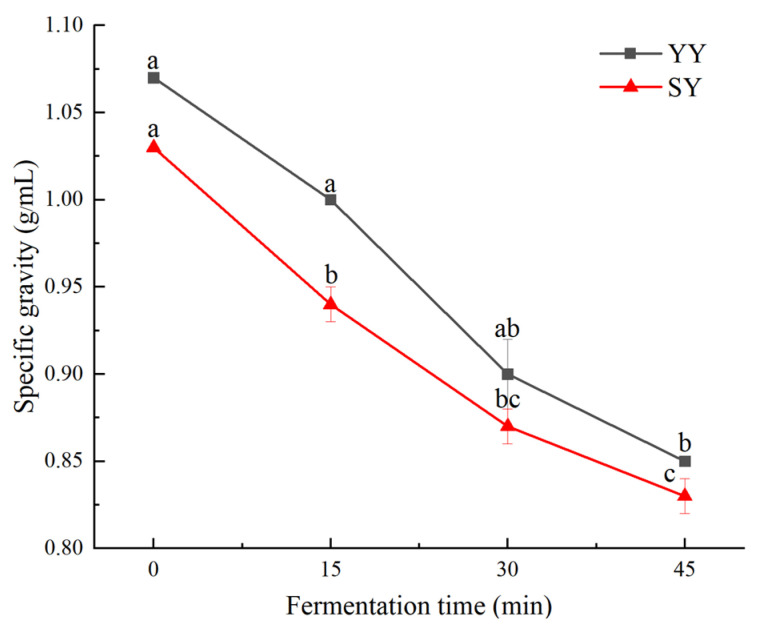
The changes in specific gravity content of corn Fagao batter. Different lowercase letters indicate significant differences between different fermentation time at the same fermentation method (*p* < 0.05).

As illustrated in Figure 4, the specific gravity of corn Fagao batter decreased as fermentation progressed. The specific gravity of the co-fermentation group was always lower than that of the yeast fermentation group. On the one hand, sourdough is a rich source of Lactic acid bacteria and its fermentation products such as extracellular polysaccharides [28]. During the fermentation process, yeast provides a range of essential nutrients, including amino acids and vitamins, which are utilized by Lactic acid bacteria for fermentation. In turn, Lactic acid bacteria facilitates the growth of yeast by supplying reducing sugars [29,30]. The fermentation of the two bacteria is mutually beneficial, resulting in increased gas production and a reduction in specific gravity. On the other hand, extracellular polysaccharides, which are fermentation products of sourdough, can increase the viscosity of corn Fagao batter and stabilize the batter bubbles, thereby increasing the gas content of the batter [18]. In the study conducted by Olojede et al. [31] on the impact of co-fermentation of yeast and *Pediococcus pentosaceus* on sorghum dough, it was observed that the structure of the dough underwent an improvement during fermentation, accompanied by an increase in its gas retention capacity. This is similar to the results of this experiment.

#### 3.1.4. Viscosity of Corn Fagao Batter

Batter viscosity is a crucial indicator of the quality of Fagao. A suitable batter viscosity facilitates the expansion and shaping of the bubbles during the maturing process to ensure that the Fagao has a high specific volume. Higher viscosity can slow down the migration and diffusion of air bubbles, which is conducive to maintaining the stability of the batter. Therefore, an appropriate increase in batter viscosity is conducive to improving the quality of the product.

**Figure 5:**
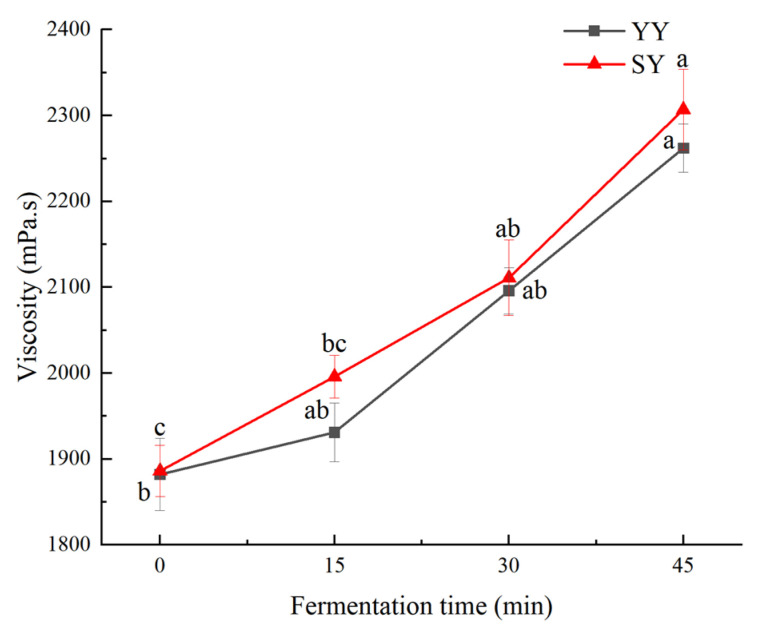
The changes in viscosity of corn Fagao batter. Different lowercase letters indicate significant differences between different fermentation time at the same fermentation method (*p* < 0.05).

As shown in Figure 5, the viscosity of the corn Fagao batter showed an upward trend with the fermentation time. This result may be attributed to the penetration of acids and enzymes produced by fermentation into the interior of starch granules, disrupting the chemical bonds between proteins, fats, and starch. This leads to the breakdown and leaching of fats and proteins. The growth of fermenting microorganisms consumes proteins, which in turn purifies the starch to some extent, increasing the batter’s viscosity. The higher viscosity of the co-fermentation group compared to the yeast fermentation group may be due to the greater number of microbial species and numbers in the co-fermentation process. Additionally, Lactic acid bacteria fermentation results in the production of polysaccharides containing hydrophilic genes in the carbon chain, which can impede the movement of water molecules and elevate the viscosity of the system. In the study performed by Xu et al. [32], they concluded that the increase in corn Fagao batter viscosity with fermentation could be attributed to the accumulation of lactic acid produced during the growth and metabolism of the complex strain.

### 3.2. Effect of Co-Fermentation on the Properties of Starch

#### 3.2.1. Effect of Co-Fermentation on Amylose Content

**Figure 6:**
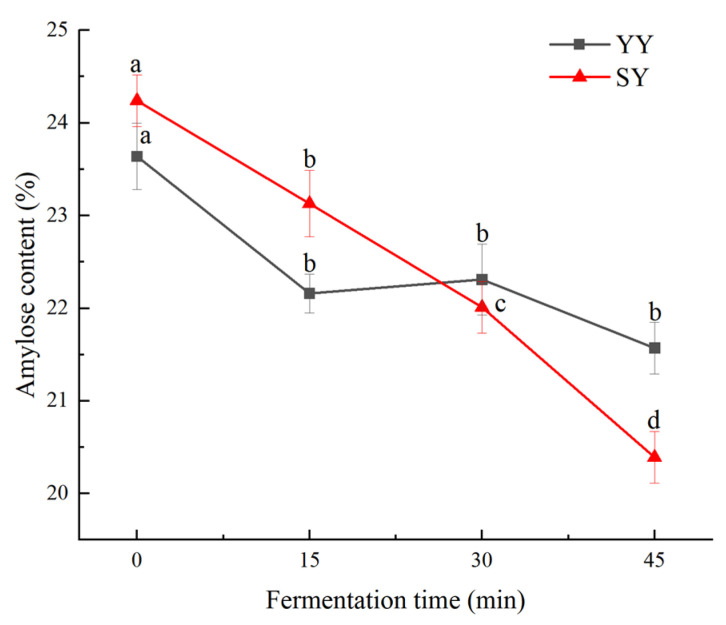
Amylose content of corn Fagao batter during fermentation. Different lowercase letters indicate significant differences between different fermentation time at the same fermentation method (*p* < 0.05).

The amylose content during the fermentation of corn Fagao batter is illustrated in Figure 6. As fermentation progressed, the amylose of corn Fagao batter in the two groups showed a downward trend. And the amylose content decreased significantly in the co-fermentation group. The co-fermentation group exhibited a higher amylose content than the yeast fermentation group during early fermentation and this might be due to the degradation of amylopectin into amylose during the fermentation of sourdough, which increases the amylose content after the addition of sourdough. As the fermentation progressed, the amylose content of the co-fermentation group was gradually lower than that of the yeast fermentation. This phenomenon may be due to the greater degradation of amylose by more acids and enzymes produced by the co-fermentation process. The decline in the amylose content during fermentation may be due to the organic acids or enzymes produced by the metabolism of the dominant microorganisms during fermentation worked on the amorphous zone, a relatively loose structure of starch, thus resulting in the dissolution of amylose [33]. In addition, the α-amylase produced by microbial metabolism degraded the amylose into small molecules such as dextrin or monosaccharides [34]. Therefore, the amylose content decreased during fermentation. In the study performed by Zhao et al. [33], they concluded that the reduction in amylose content of wheat starch with fermentation time is due to the metabolites produced by the fermenting microorganisms breaking down the starch into small-molecule sugars and thus obtaining the carbon and energy sources needed for growth and reproduction.

#### 3.2.2. Effect of Co-Fermentation on the Microscopic Structure of Starch

**Figure 7:**
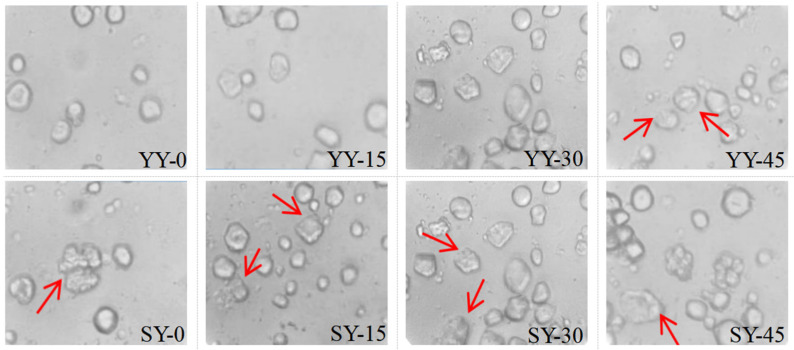
Optical micrograph of starch granules during fermentation. The arrows show the locations where alterations in starch granules have been observed.

Changes in the microscopic morphology of corn starch during fermentation can explain the effect of different fermentation times on the physicochemical and functional properties. As shown in Figure 7, corn starch granules exhibit irregularly rounded or polygonal. The starch granules were essentially unchanged during the early fermentation of the yeast. Corn starch granules in the co-fermentation group had disrupted and eroded during the early fermentation, which was likely due to the sourdough itself. In the yeast fermentation group, at 45 min, the starch granules began to lose their distinct boundaries, erosion and cracks occurred more extensively on the granule surface, and small holes appeared, leading to the formation of porous network structures. This phenomenon manifested at 30 min in the co-fermentation group. The change may be attributed to the hydrolysis of the starch granules by acid and amylase, which are produced during the fermentation process. This results in pores, erosion, and cracks on the surface of the starch granules [35]. Furthermore, the fermentation process results in the production of numerous proteases, which facilitate the separation of starch and protein and the formation of holes [33]. The co-fermentation group may produce more enzymes and show a stronger hydrolyzation towards starch molecules. In general, fermentation destroys starch granules, a phenomenon that becomes more and more apparent as fermentation proceeds. This disruption provides a channel for substances such as water molecules to enter the interior of the starch granules, altering the internal crystalline regions of the granules and thus affecting the physicochemical properties of the starch.

#### 3.2.3. Effect of Co-Fermentation on the Crystalline Structure of Starch

**Figure 8:**
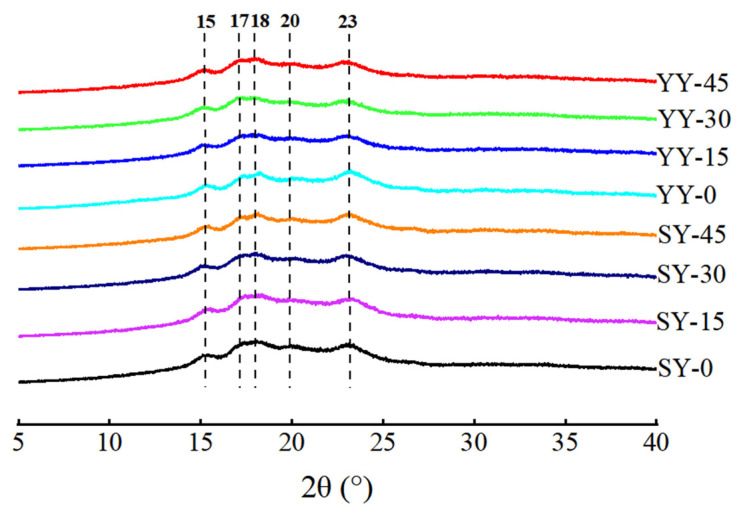
XRD patterns of corn starch granules during fermentation.

Starch granules are a semi-crystalline biopolymer consisting of crystalline and amorphous regions. The crystalline structure of starch can be classified into A, B, C, and V types based on the characteristic peaks of the XRD pattern. The crystalline structures of corn starch are shown in Figure 8. All corn starches of different fermentation conditions showed A + V hybrid crystalline structures with diffraction peaks at 15, 17, 18, 23° (2θ) (A-type), and 20° (2θ) (V-type) [36]. Microorganism fermentation did not change starch crystalline type, as no new diffraction peaks appeared. Starch hydrolysis by fermentation consists mainly of acid hydrolysis and enzymatic hydrolysis, which hydrolyses the glycoside bonds of starch. The A-type crystalline form is thermally stable, and fermentation is not sufficient to change the crystalline form of starch [37]. Similar results were reported by Zhao et al. [33], showing that the impact of microbial fermentation on starch is predominantly concentrated within the amorphous zone and is insufficient to alter the crystalline structure of the starch.

**Figure 9:**
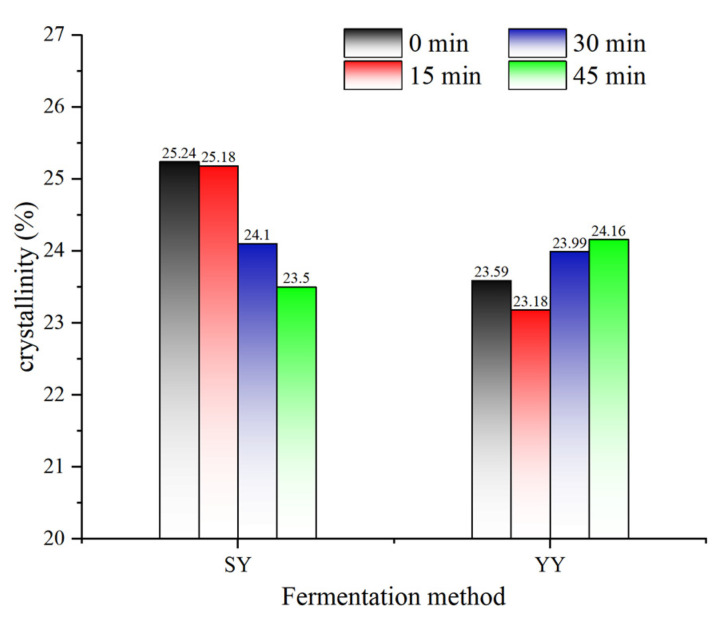
Changes in starch crystallinity of corn Fagao batter with different fermentation.

Native starch granules are partially crystalline, exhibiting a degree of crystallinity that typically ranges from 15% to 45%. The amorphous regions of the starch granules are composed mainly of amylose chains and the branching point regions of the amylopectin chains. The crystalline regions are composed of double helices packed into crystalline lattices [38]. The change in the relative crystallinity of corn starch during fermentation is presented in Figure 9. A slight increase in the crystallinity of yeast fermentation may be attributed to the amorphous regions of starch being structurally unstable and susceptible to degradation. Yeast fermentation has been observed to preferentially hydrolyze the amorphous regions, resulting in an increase in crystallinity [34]. The relative crystallinity of corn starch during fermentation showed an upward trend. The hydrolysis of starch by a multitude of acids and enzymes produced by microbial metabolism in sourdough was enhanced. The acids and enzymes penetrate the starch granules, where they hydrolyze the long amylopectin in the crystalline area of the starch granules. This process generates a large number of intermediate and short amylose starches, which alter the molecular structure of starch and result in a reduction in the crystallinity of starch [24].

#### 3.2.4. Effect of Co-Fermentation on the Pasting Properties of Starch

**Figure 10:**
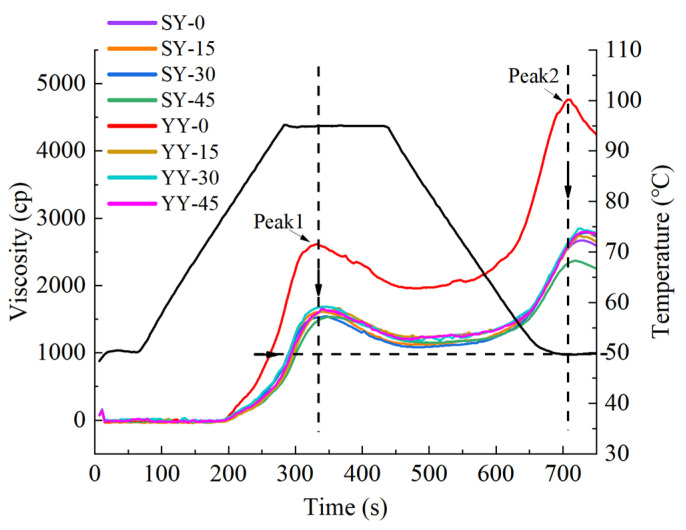
Pasting properties curve of corn starches with different fermentation methods.

The pasting properties of starch are largely related to the quality of the starch food and are regarded as a crucial parameter to evaluate the quality of grain food. As described in Figure 10, fermentation shifted the pasting curve overall downward and rightward at the same time, and the gelatinized temperature increased. Peak 1 (peak viscosity) and peak 2 (setback value) were observed to occur simultaneously in the fermented and non-fermented corn Fagao batter pasting curves. However, the peaks of the fermented corn Fagao batter were all greatly reduced. It shows that fermented corn flour is not easy to gelatinization and the viscosity is reduced after gelatinization. Products manufactured using fermented corn Fagao batter are more resistant to aging, especially with co-fermentation.

The pasting parameters of different fermentation conditions starch are shown in Table 1. Peak viscosity, through viscosity, final viscosity, and setback value decreased as fermentation progressed. Furthermore, the pasting viscosity of the co-fermentation group was lower than that of the yeast fermentation group. The pasting viscosity of starch is mainly influenced by the solubilization of amylose, the length of amylopectin branches, and the swelling characteristics of starch granules [39]. The decrease in pasting viscosity has the following reasons. On the one hand, fermentation results in the hydrolysis of proteins and lipids encapsulated outside the starch, allowing the amylose molecules to come out of the starch granules; on the other hand, fermentation results in the production of a considerable quantity of enzymes and acids, which facilitate the hydrolysis of α-1,4 and α-1,6 glycoside bonds in starch and reduce the spatial site resistance of the starch molecules [34]. They reported that the process of fermentation results in the hydrolysis of the side chains of branched starch, which leads to a reduction in the pasting viscosity [15]. The breakdown value is indicative of the capacity of starch to withstand mechanical shear during the heating process. A lower breakdown value is indicative of the enhanced stability of the starch granules. The setback value is indicative of the stability and retrogradation tendency of starch following gelatinization. A smaller setback value is indicative of a lighter degree of re-crystallization of starch following gelatinization. The breakdown and setback values depend on the extent of change in viscosity subsequent to starch gelatinization. Factors affecting this include the amylose content, the length of the starch molecular chain, and the molecular weight of the starch [40]. The setback and breakdown values were found to be reduced for both fermentation methods, indicating that fermentation results in increased thermal stability and resistance to aging of corn Fagao batter. Furthermore, the co-fermentation group was observed to perform better. This result is in agreement with the results of amylose content, microstructure, and crystallinity of the corn Fagao batter. Similar results were reported by Oyarekua et al. [41]. Additionally, the pasting properties of maize flour exhibited differences across the various corn varieties.

#### 3.2.5. Effect of Co-Fermentation on the Short-Range Ordered Structure of Starch

**Figure 11:**
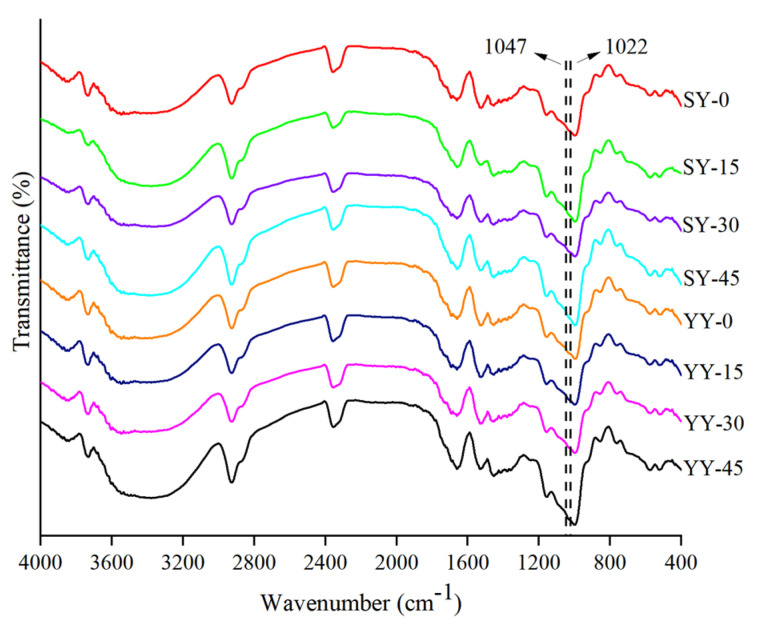
FTIR diffraction patterns of corn starches with different fermentation methods.

The short-range ordered structure of starch refers to the structure formed by the orderly stacking of short distances between double helixes, which can reflect the degree of internal structural orderliness of the starch chains. In the infrared spectrogram of starch (Figure 11), the different bands of 400–4000 cm^−1^ correspond to the different chemical bonds of the starch. The absorption peaks at 2800–3000 cm^−1^ were attributed to the C-H stretching vibration, the absorption peaks at 3000–3600 cm^−1^ were assigned to the O-H of starch stretching vibration absorption of the O-H, the absorption peaks at 900–1300 cm^−1^ were attributed to C-O, C-C, and C-O-H stretching as well as C-O-H bending vibration, and the absorption peaks between this region were the sensitive bands for starch conformation. The absorption peaks at 1047 cm^−1^ were sensitive to the molecular crystalline structure and the band at 1022 cm^−1^ was linked to the amorphous structure of starch [42,43]. The ratio of the integrated area of the absorption bands at 1047/1022 cm^−1^ was generally used to quantify the internal changes of the starch molecule in the degree of short-range order, respectively. The short-range ordered structure had a positive correlation with the 1047/1022 cm^−1^ ratio [44].

As shown in Table 2, as fermentation progressed under different fermentation conditions, the 1047/1022 cm^−1^ ratio showed a decreasing tendency, suggesting that ultrasound irradiation might act on starch by weakening the short-range crystallinity. The enzymes produced during fermentation break down large starch molecules, disrupting the crystalline structure of starch and producing short-chain starch molecules, which results in a decrease in the orderliness of starch. The 1047/1022 cm^−1^ ratio in the co-fermentation group exhibited a notable decline from 0.962 to 0.950, which was a more pronounced reduction in orderliness than that observed under yeast fermentation. This finding suggests that co-fermentation may disrupt the ordered structure of starch to a greater extent than yeast fermentation. Similar results were reported by Qi et al. [45].

### 3.3. Effect of Co-Fermentation on the Relative Molecular Weight of Proteins

Corn proteins can be divided into zein, glutenin, globulin, and albumin. Zein is the predominant protein in corn, comprising 60–70% of the total protein content. The structural function of zein is analogous to that of glutenin, which enables it to form a viscoelastic network [46]. Zein proteins are classified into four protein subunits, α-zein, β-zein, γ-zein, and δ-zein, based on their molecular weight, solubility, and charge. The predominant subunit is α-zein, with a molecular weight of 21–25 kDa, followed by β-zein, with a molecular weight of 15–17 kDa, γ-zein, with a molecular weight of 27 kDa, and a small portion of δ-zein [47]. Protein distribution has a great influence on the structure, physicochemical properties, and rheological properties of starch [48].

**Figure 12:**
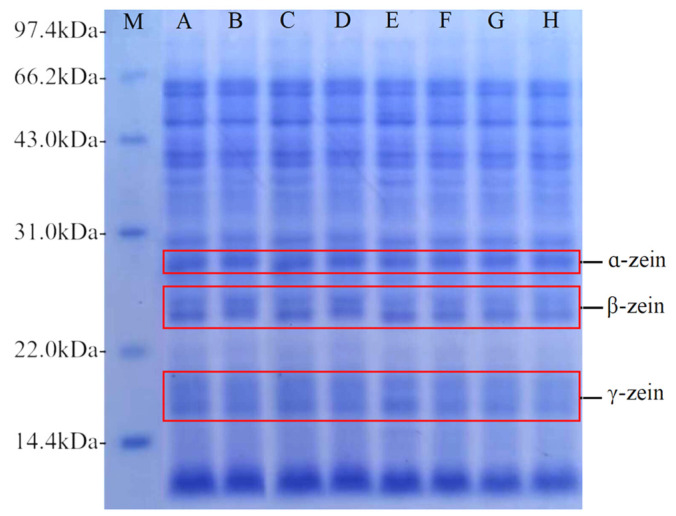
Gel electrophoresis of corn protein with different fermentation methods. M represents low molecular weight marker; A, B, C, and D represent samples of yeast fermented for 0, 15, 30, and 45 min; E, F, G, and H represent samples of co-fermented with sourdough and yeast for 0, 15, 30, and 45 min.

**Figure 13:**
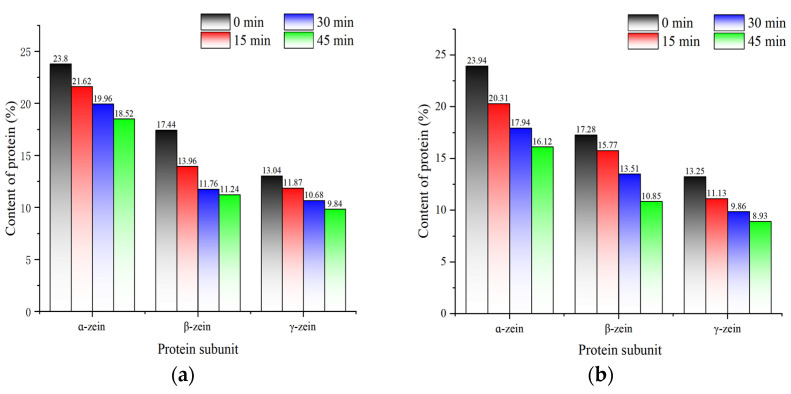
The change in α-, β-, and γ- zein content in different fermentation methods. (**a**) Changes in the content of protein subunits during yeast fermentation; (**b**) Changes in the content of protein subunits during co-fermentation with sourdough and yeast.

The bands of each subunit of corn protein can be clearly seen from Figure 12. The molecular weights corresponding to each subunit were mainly concentrated in the range of 12.03 kDa, 16.64 kDa, 23.97 kDa, 28.33 kDa, and 36.78–86.11 kDa. As fermentation progressed, the number of subunit bands remained unaltered, but the area occupied by each subunit band exhibited a varying degree of reduction. Figure 13 shows the results of the quantitative analysis of α-zein, β-zein, and γ-zein by Quantity One. At the beginning of fermentation, the contents of α-, β-, and γ-zein were similar between the two fermentation conditions. As fermentation progressed, there was a decline in the contents of each subunit, with co-fermentation resulting in a more pronounced reduction in protein subunit contents than yeast fermentation. Proteases activied during fermentation help hydrolyzing proteins to produce small molecules such as peptides, which are a source of nitrogen for the growth and metabolism of the strain. This process results in a reduction in corn protein content during fermentation [49]. In contrast, synergistic fermentation leads to the production of more proteases. The results of this analysis were consistent with the results of the viscosity of corn Fagao batter and its microscopic structure.

## 4. Conclusions

In this study, alterations in the physicochemical properties of the corn Fagao batter were examined during the sourdough–yeast co-fermentation. Compared with the control sample, the sourdough–yeast co-fermentation enhanced the acid-producing capacity, gas production, and viscosity of corn Fagao batter, which may contribute to the volume and flavor of corn Fagao batter. In contrast, the co-fermented batter shows a more pronounced increase in reducing sugar content, higher hydrolysis of starch, and less amylose content. During the fermentation process, the starch granules lost their distinct boundaries, erosion and cracks occurred more extensively on the granule surface, and small holes appeared, leading to the integrity of the starch granules being damaged. This phenomenon manifested more seriously in the co-fermentation group. The crystallinity was less than the yeast-fermented batter, even though the crystal structure type of starch did not obviously change. Fourier Transform infrared analysis indicated that the 1047/1022 cm^−1^ ratio in the co-fermentation group exhibited a notable decline from 0.962 to 0.950, which suggests that co-fermentation may disrupt the ordered structure of starch to a greater extent than yeast fermentation. The peak viscosity, minimum viscosity, final viscosity, decay value, and recovery value of corn Fagao batter are reduced by co-fermentation, which can improve the thermal stability of corn Fagao batter and slow down the aging. Co-fermentation also results in a more pronounced reduction in protein subunit content than yeast fermentation. In conclusion, co-fermentation can promote the hydrolysis of starch and protein as well as change the crystallinity of starch. This may improve the sensory quality of the Fagao product and delay the staling process. The results of this study not only explained the improvement in the quality of corn Fagao made from the co-fermentation method but also offered theoretical references for co-fermentation with sourdough and yeast to other corn-based fermented foods.

## Figures and Tables

**Table 1:** Pasting properties of corn starches with different fermentation methods.

	Peak Viscosity/cp	Through Viscosity/cp	Breakdown/cp	Final Viscosity/cp	Setback/cp	Pasting Temperature/°C
SY-0	1632	1228	404	2441	1213	77.4
SY-15	1612	1121	491	2556	1435	78.35
SY-30	1542	1082	460	2572	1490	77.5
SY-45	1538	1146	392	2120	974	78.25
YY-0	2616	1958	658	4044	2086	76.65
YY-15	1665	1225	440	2512	1287	79.05
YY-30	1687	1184	503	2605	1421	63.65
YY-45	1653	1196	457	2624	1428	66.8

**Table 2:** The short-range ordered structure of corn starches with different fermentation methods. Different lowercase letters indicate significant differences between different fermentation time at the same fermentation method (*p* < 0.05).

Sample	1047/1022 cm^−1^	Sample	1047/1022 cm^−1^
SY-0	0.962 ± 0.004 ^ab^	YY-0	0.965 ± 0.005 ^a^
SY-15	0.955 ± 0.001 ^ab^	YY-15	0.962 ± 0.007 ^ab^
SY-30	0.953 ± 0.007 ^ab^	YY-30	0.960 ± 0.003 ^ab^
SY-45	0.950 ± 0.004 ^b^	YY-45	0.956 ± 0.000 ^ab^

## Data Availability

The original contributions presented in this study are included in the article. Further inquiries can be directed to the corresponding author.
